# Safety and Glycemic Outcomes Among Youth With New‐Onset Type 1 Diabetes Using a Tubeless Automated Insulin Delivery System

**DOI:** 10.1155/jdr/2896084

**Published:** 2026-05-04

**Authors:** Daniel J. DeSalvo, Natalie M. Linde, James Sickler, Carolina Villegas, Marzia Cescon

**Affiliations:** ^1^ Department of Pediatrics, Baylor College of Medicine, Houston, Texas, USA, bcm.edu; ^2^ Department of Mechanical and Aerospace Engineering, University of Houston, Houston, Texas, USA, uh.edu

## Abstract

**Introduction:**

Automated insulin delivery (AID) systems aim to safely optimize glycemic outcomes; however, data in the new‐onset type 1 diabetes (T1D) period are limited. We evaluated safety and glycemic outcomes of Omnipod 5 AID System use in the new‐onset period.

**Methods:**

This was a retrospective observational analysis of youth at a single center who initiated Omnipod 5 within 90 days of T1D diagnosis. Safety outcomes included diabetic ketoacidosis (DKA) and severe hypoglycemia. Continuous glucose monitoring (CGM) metrics and insulin delivery data were analyzed across the first 3 months of use and between pediatric age groups (<6‐year‐olds, 6 to <12‐year‐olds, and 12 to <18‐year‐olds).

**Results:**

Among 74 youth initiating Omnipod 5 within 90 days of T1D diagnosis, there was no occurrence of DKA or severe hypoglycemia in the first 3 months of AID system use. The time‐weighted average glucose target value on the device setting was 118.8 mg/dL (6.6 mmol/L) with a median of 97% time in Automated Mode over 3 months. Median total daily insulin was 7.4 units (45.5% basal) in <6‐year‐olds, 11.4 units (48% basal) in 6 to <12‐year‐olds, and 25.7 units (45% basal) in 12 to <18‐year‐olds. The median glucose management indicator (GMI) was 7.1% (54 mmol/mol) and time in range (70–180 mg/dL, 3.9–10.0 mmol/L) was 72.1% with minimal hypoglycemia: 1.0% Level 1 time below range (TBR, 54–69 mg/dL, 3.0–3.8 mmol/L) and 0% Level 2 TBR (<54 mg/dL, <3.0 mmol/L).

**Conclusion:**

These real‐world observational data from youth with new‐onset T1D using a tubeless AID system indicate safety and favorable glycemic outcomes with AID system initiation early after diagnosis. Future studies should assess the long‐term glycemic and quality of life impact of early AID system adoption.

## 1. Introduction

The period immediately following a diagnosis of type 1 diabetes (T1D) represents a critical window during which families must rapidly acquire diabetes management skills while navigating significant physiologic changes associated with the onset of insulin therapy. Early glycemic patterns during this phase have been shown to influence long‐term metabolic outcomes, as intensive glycemic control initiated soon after diagnosis is associated with reduced risk of microvascular and macrovascular complications later in life [1]. Automated insulin delivery (AID) systems aim to safely optimize glycemic outcomes while alleviating the burden of complex diabetes management [2], which may be especially beneficial for newly diagnosed youth and their families. However, data on AID use in the new‐onset T1D period remain limited, underscoring the need for real‐world evidence to inform clinical practice.

At Texas Children’s Hospital (TCH)—a large, tertiary children’s health system with a diverse patient population—we have a clinical program supporting newly diagnosed youth with T1D and their families to initiate CGM within 1–2 days of diagnosis and provide the opportunity to initiate AID systems soon after diagnosis. In the first outpatient visit, which usually occurs 2–4 weeks after T1D diagnosis, patients and families are routinely educated on AID systems. Each of the commercially available AID systems is presented in detail to the family, who can choose which, if any, system they would like to initiate. The Omnipod 5 AID System is available through the pharmacy channel, which expedites the process of obtaining, training, and initiating the AID system early in the new‐onset T1D period. In this study, we aimed to evaluate glycemic outcomes and safety data, including occurrence of diabetic ketoacidosis (DKA) and severe hypoglycemia, in children who initiated the Omnipod 5 AID System within the first 3 months of diagnosis at our center.

In pivotal trials, Omnipod 5 was safe and effective in people with established T1D age 2–70 years, with an increase in time in range (TIR, 70–180 mg/dL, 3.9–10 mmol/L) from baseline therapy and low levels of hypoglycemia with a median time below range (TBR, <70 mg/dL, <3.0 mmol/L) below 3% for all age groups [3, 4]. Given this clinical context, understanding how this AID system performs when initiated shortly after T1D diagnosis is an important next step. In this study, we evaluated the real‐world safety and clinical outcomes with Omnipod 5 AID System use in the new‐onset T1D period.

## 2. Methods

This was a retrospective observational analysis of youth (<18 years old) with new‐onset T1D who initiated the Omnipod 5 AID System within 90 days of diabetes diagnosis at TCH, beginning with the initial FDA clearance of Omnipod 5 in January 2022 through December 2023. Omnipod 5 includes a tubeless insulin pump (“Pod”) with an embedded dosing algorithm that adjusts insulin delivery every 5 min based on current and predicted glucose values [5]. Glucose targets are set at 110–150 mg/dL (6.1–8.3 mmol/L) in 10 mg/dL (0.55 mmol/L) increments, adjustable by time of day with up to eight different allowable time segments. The Omnipod 5 App is used to adjust settings, deliver boluses, and turn Automated Mode on/off. The Activity feature can be used to constrain insulin delivery during periods of hypoglycemia risk. Omnipod 5 device data are automatically uploaded to Glooko’s cloud‐based data management system via SIM card or wireless internet, which removes the need for manual upload and makes data easily available for review.

Inclusion criteria for this study included T1D diagnosis confirmed by the presence of ≥1 islet autoantibody, age <18 years, initiation of Omnipod 5 within 3 months of diabetes diagnosis with verifiable data in Glooko, and consistent use of CGM (>80% use). Omnipod 5 users who only used “Manual Mode” without any use of “Automated Mode” in the initial 3 months were excluded from the analysis. Sociodemographic data, including age at T1D diagnosis, gender, self‐reported race and ethnicity, hemoglobin A1c (HbA1c), and presence of DKA at time of T1D diagnosis, were collected from the electronic health record (EHR). The exact start date of Omnipod 5, including use of Automated Mode, was verified on a PDF‐generated report in Glooko, and all data were summarized according to Month 1 (Days 1–30), Month 2 (Days 31–60), Month 3 (Days 61–90), and aggregate (Days 1–90). Omnipod 5 device pattern use, including Target Glucose settings and insulin delivery data (i.e., total daily insulin dose, % basal, % bolus, % time in Automated Mode, Activity feature, and Manual Mode), was obtained from Glooko along with glycemic data, including glucose management indicator (GMI), mean glucose, coefficient of variation (CV), TIR (70–180 mg/dL, 3.9–10.0 mmol/L), Level 1 time above range (TAR, 181–250 mg/dL, 10.1–13.9 mmol/L), Level 2 TAR (>250 mg/dL, >13.9 mmol/L), Level 1 TBR (54–69 mg/dL, 3.0–3.8 mmol/L), and Level 2 TBR (<54 mg/dL, <3.0 mmol/L). The average glucose target setting for each time segment in the Glooko report was calculated as a time‐weighted average and grouped by rounding to the nearest of the five available Target Glucose Settings (i.e., 110, 120, 130, 140, or 150 mg/dL; 6.1, 6.7, 7.2, 7.8, or 8.3 mmol/L). GMI was used in this study as a calculated estimate of laboratory‐measured HbA1C [6] since HbA1c was not routinely available at the key study time points. Data on safety outcomes, including DKA and severe hypoglycemia, were extracted from the EHR for the first 3 months following AID system initiation. Any hospital visits for DKA or severe hypoglycemia occurring at outside hospital systems are recorded on a provider flowsheet, so this was available in our EHR. The definition of DKA was based on American Diabetes Association (ADA) criteria, including pH <7.3 and/or bicarbonate <15 mmol/L [7].

The study was approved by the Baylor College of Medicine Institutional Review Board and granted a waiver of consent. Study data were collected, managed, and securely stored using REDCap electronic data capture tools.

### 2.1. Statistical Analysis

Summary data are reported as median and interquartile range for continuous data or frequency and percentage for categorical data. The Wilcoxon signed‐rank test was used to compare glycemic outcomes across each of the initial 3 months of AID system use in the new‐onset period. Glycemic data were compared between age groups by the Kruskal–Wallis rank sum test. In an exploratory analysis, the Wilcoxon rank sum test was used to compare CGM metrics between those using an average target of 110 mg/dL (6.1 mmol/L) versus other targets. Statistical analyses were conducted using MATLAB (2023a), Natick, Massachusetts: The MathWorks, Inc. Summary data are presented as median (interquartile range) or mean ± standard deviation. *p*‐Values with a two‐sided significance level of 0.05 were considered significant.

## 3. Results

Among 74 youth with new‐onset T1D who initiated Omnipod 5 within 3 months of diagnosis, the average age was 9.5 ± 4.0 years (range 1.3–16.6 years) at time of diagnosis; 53.2% were female; and race/ethnicity included 83.6% non‐Hispanic white, 4.1% non‐Hispanic black, 6.8% Hispanic, and 5.5% Asian. At the time of T1D diagnosis, mean HbA1c was 11.6 ± 2.2%, and 34 (45.9%) presented in DKA. The sociodemographic and clinical characteristics by age group and overall cohort are displayed in Table 1.

**Table 1:** Sociodemographic and clinical characteristics by age group and overall cohort.

Age group	<6 years (*N* = 16)	6 to <12 years (*N* = 37)	12 to <18 years (*N* = 21)	Total sample (*N* = 74)
Age (years)	3.8 ± 1.2	9.3 ± 1.7	14.2 ± 1.4	9.5 ± 4.0
Female sex, *N* (%)	9 (56.2%)	20 (54.1%)	10 (47.6%)	39 (53.2%)
Race/ethnicity, *N* (%)				
Non‐Hispanic white	13 (81.2%)	31 (83.8%)	18 (85.7%)	62 (83.8%)
Non‐Hispanic black	2 (12.5%)	1 (2.7%)	0 (0.0%)	3 (4.1%)
Hispanic	0 (0.0%)	4 (10.8%)	1 (4.8%)	5 (6.8%)
Asian	1 (6.2%)	1 (2.7%)	2 (9.5%)	4 (5.4%)
HbA1c at diagnosis (%)	10.3 ± 2.2	11.6 ± 2.0	12.5 ± 2.0	11.6 ± 2.2
DKA at diagnosis, *N* (%)	8 (50.0%)	15 (40.5%)	11 (52.4%)	34 (45.9%)

The average time to Omnipod 5 initiation was 47 ± 18 days after T1D diagnosis. There were no episodes of DKA or severe hypoglycemia during the first 3 months of tubeless AID system use in the new‐onset T1D period.

### 3.1. Device Settings

**Figure 1:**
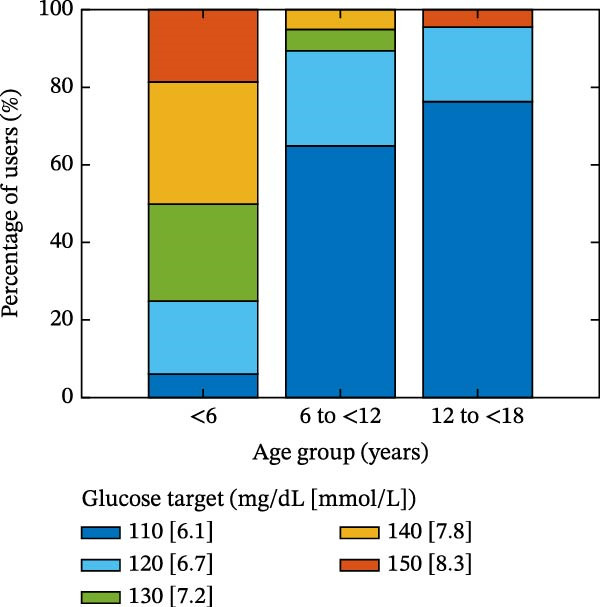
Glucose target use patterns showing the percentage of each time‐weighted average glucose target level rounded to the nearest setting value by age group. The stacked bar graph displays targets in descending order from top to bottom.

The time‐weighted average glucose target value across the aggregate 3‐month period of Omnipod 5 AID System use was 118.8 mg/dL (6.6 mmol/L) across all users. There were differences in average glucose targets used across age, with the youngest group (<6 years old) using higher settings (shown in Figure 1). In the youngest group of <6‐year‐olds, 75.0% had an average target of 130–150 mg/dL (7.2–8.3 mmol/L), compared to 10.8% of 6 to <12‐year‐olds and 4.8% of 12 to <18‐year‐olds. Among all youth with new‐onset T1D, 55.4% had an average target of 110 mg/dL (6.1 mmol/L), including 6.2% in the youngest group, compared to 64.9% in 6 to <12 years and 76.2% in 12 to <18 years. As shown in Figure 2, during the first 3 months of AID system use in the new‐onset T1D period, the time‐weighted average glucose target decreased over time in the youngest group but remained higher than the older groups, even in Month 3.

Figure 2Time‐weighted average glucose target in months 1–3 in the new‐onset T1D period for each of the age groups. In the box plots, horizontal bars represent the median, the lower and upper boundaries of each box represent the 25^th^ and 75^th^ percentiles, and the plus signs represent outliers calculated by Q3 + 1.5  ^∗^ (Q3–Q1) for the upper fence and Q1−1.5  ^∗^ (Q3–Q1) for the lower fence. (a) <6 years old, (b) 6 to <12 years old, and (c) 12 to <18 years old.

**Figure:**
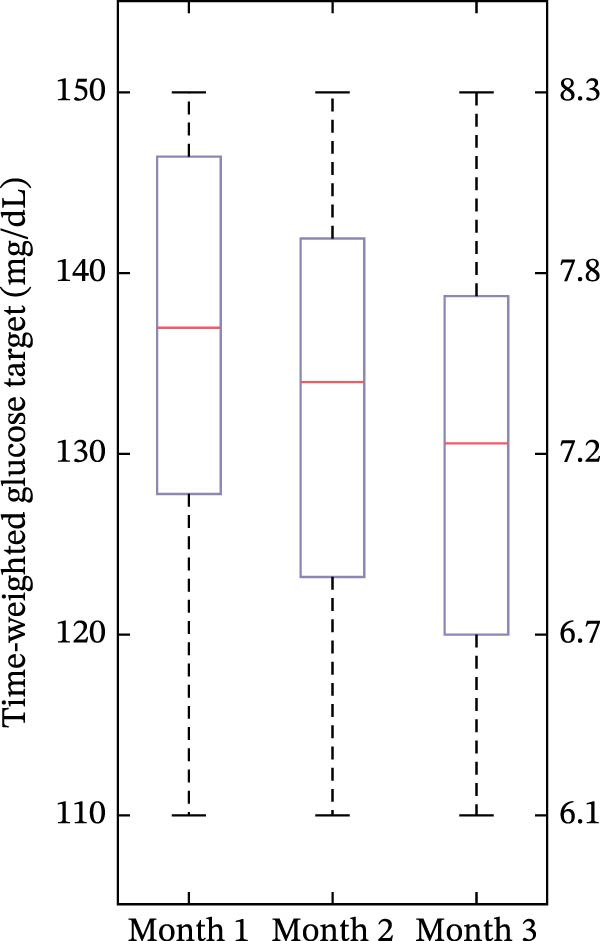
(a)

**Figure:**
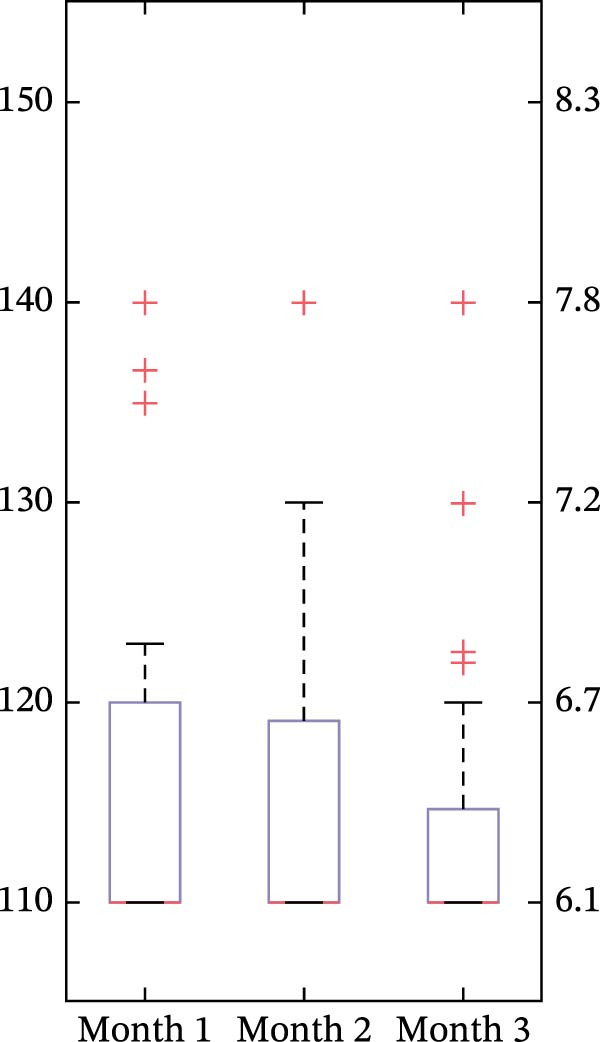
(b)

**Figure:**
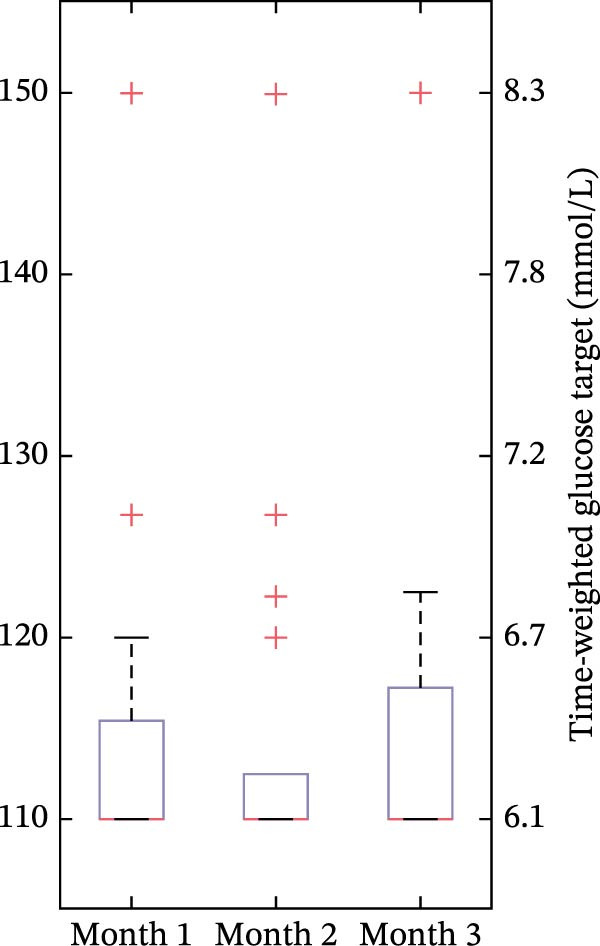
(c)

### 3.2. Insulin Delivery Data

Insulin delivery data with Omnipod 5 across the 3‐month new‐onset period are available in Table 2. The median percent time in Automated Mode over the aggregate 3 months was 97 [93, 99]%, including 98 [96, 100]% in Month 1, 98 [93, 100]% in Month 2, and 97 [90, 100]% in Month 3. There was no difference in time spent in Automated Mode across age groups (*p* = 0.06). The median percent time using the Activity feature was 1 [0, 1]% across the aggregate 3‐month period, including 1 [0, 1]% in <6‐year‐olds, 1 [0, 1]% in 6 to <12‐year‐olds, and 0 [0, 2.5]% in 12 to <18‐year‐olds. The median total daily insulin across the 3‐month period of AID system use was 7.4 units/day in <6‐year‐olds (7.3 units/day in Month 1, 7.2 units/day in Month 2, 8.1 units/day in Month 3), 11.4 units/day in 6 to <12‐year‐olds (10.8 units/day in Month 1, 11.5 units/day in Month 2, 12.6 units/day in Month 3), and 25.7 units/day in 12 to <18‐year‐olds (25.6 units/day in Month 1, 27.2 units/day in Month 2, 27.4 units/day in Month 3). The median number of user‐initiated boluses per day was 4.7 [3.6, 5.7] overall, including 5.3 [3.5, 6.2] in <6‐year‐olds, 4.5 [3.4, 5.5] in 6 to <12‐year‐olds, and 4.7 [3.8, 6.0] in 12 to <18‐year‐olds. Rates of overriding the bolus calculator were similar across age groups, including 4 [0.5, 18.5]% in the youngest group, 2 [0.0, 12.5]% in 6 to <12‐year‐olds, and 3 [0, 19]% in 12 to <18‐year‐olds (*p* = 0.4).

**Table 2:** Real‐world outcomes among age groups in the aggregate 3 months of Omnipod 5 AID system use in the new‐onset T1D period.

Age group	<6 years (*N* = 16)	6 to <12 years (*N* = 37)	12 to <18 years (*N* = 21)	All ages (<18 years, *N* = 74)	*p*‐Value
GMI (%) GMI (mmol/mol)	7.6 ± 0.6, 7.5 [7.2, 7.8] 59.7 ± 6.3, 58.5 [55.2, 61.7]	7.0 ± 0.5, 7.1 [6.7, 7.3] 53.9 ± 4.9, 54.1 [49.7, 56.3]	6.8 ± 0.4, 6.8 [6.4, 7.0] 51.2 ± 4.2, 50.8 [46.4, 53.0]	7.1 ± 0.6, 7.1 [6.7,7.4] 53.9 ± 6.0, 54.1 [49.7, 57.4]	*p* < 0.001
*N* (%) achieving ADA target^a^	3 (18.8%)	16 (43.2%)	16 (76.2%)	35 (47.3%)	—

Time in glucose ranges					
Level 2 time below range (%) <54 mg/dL [<3.0 mmol/L]	0.06 ± 0.25, 0.0 [0.0, 0.0]	0.03 ± 0.16, 0.0 [0.0, 0.0]	0.0 ± 0.0, 0.0 [0.0, 0.0]	0.03 ± 0.16, 0.0 [0.0, 0.0]	*p* = 0.51
Level 1 time below range (%) 54–69 mg/dL [3.0–3.8 mmol/L]	0.7 ± 0.6, 1.0 [0.0, 1.0]	0.6 ± 0.6, 1.0 [0.0, 1.0]	0.5 ± 0.5, 0.0 [0.0, 1.0]	0.6 ± 0.6, 1.0 [0.0, 1.0]	*p* = 0.57
Time in range (%) 70–180 mg/dL [3.9–10.0 mmol/L]	57.8 ± 11.9, 57.0 [52.5, 65.5]	73.7 ± 12.0, 71.0 [65.8, 80.5]	80.1 ± 10.4, 80.0 [74.8, 90.3]	72.1 ± 14.0, 72.5 [64.0, 82.0]	*p* < 0.001
Level 1 time above range (%) 181–250 mg/dL [10.1–13.9 mmol/L]	24.8 ± 3.9, 25.0 [22.5, 28.0]	18.9 ± 7.3, 21.0 [15.5, 24.0]	15.3 ± 6.5, 16.0 [8.8, 19.3]	19.2 ± 7.2, 21.0 [14.0, 25.0]	*p* < 0.001
Level 2 time above range (%) >250 mg/dL [>13.9 mmol/L]	16.6 ± 9.8, 15.0 [10.5, 19.5]	6.9 ± 5.8, 6.0 [2.8, 9.3]	4.1 ± 5.1, 2.0 [1.0, 5.5]	8.2 ± 8.0, 6.5 [2.0, 12.0]	*p* < 0.001

Glucose variability					
Coefficient of variation (%)	37.0 ± 3.0, 37.2 [34.1, 39.8]	32.0 ± 4.9, 32.7 [28.8, 34.8]	29.7 ± 5.2, 28.2 [25.5, 32.6]	32.4 ± 5.3, 32.8 [28.3, 35.9]	*p* < 0.001

Insulin delivery data					
Total daily insulin (units/day)	8.1 ± 2.6, 7.4 [6.1, 9.4]	15.4 ± 10.6, 11.4 [7.9, 19.5]	29.7 ± 16.7, 25.7 [17.2, 39.9]	17.9 ± 14.0, 12.4 [7.9, 24.7]	*p* < 0.001
Proportion of insulin from boluses (%)	54.5 ± 7.8, 54.5 [49.0, 59.5]	51.4 ± 11.8, 52.0 [41.8, 57.2]	53.8 ± 9.4, 55.0 [49.8, 58.8]	52.7 ± 10.4, 53.5 [46.0, 58.0]	*p* = 0.41
Time in automated mode (%)	93.1 ± 6.2, 93.5 [90.5, 98.5]	94.8 ± 5.1, 97.0 [92.0, 98.2]	96.7 ± 5.1, 98.0 [96.0, 100.0]	95.0 ± 5.4, 97.0 [93.0, 99.0]	*p* = 0.06

*Note:* Glycemic metrics were compared between age groups by Kruskal–Wallis rank sum test.

^a^ADA target: <7%, <53 mmol/mol.

### 3.3. Glycemic Outcomes

**Figure 3:**
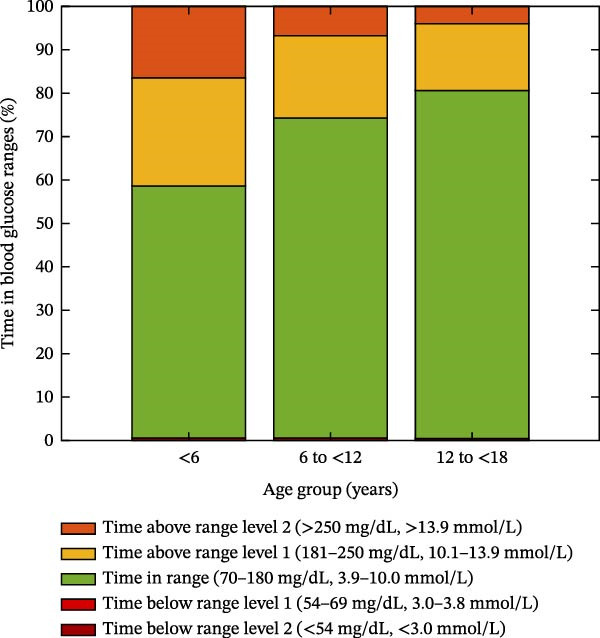
Visual representation of time in glucose ranges in the initial 3 months of Omnipod 5 AID System use in the new‐onset T1D period for each age group.

The real‐world glycemic outcomes in the initial 3 months of Omnipod 5 AID System use in the new‐onset period are displayed across age groups in Table 2. Across all users in the aggregate 3‐month period of AID system use, the median GMI was 7.1 [6.7, 7.4]%, including 6.9 [6.6, 7.4]% in Month 1, 7.0 [6.7, 7.4]% in Month 2, and 7.2 [6.8, 7.4]% in Month 3. TIR (70–180 mg/dL, 3.9–10.0 mmol/L) over the 3‐month period was 72.1 [64.0, 82.0]%. The percent time in hypoglycemia was low, including 1.0 [0.0, 1.0]% Level 1 TBR (54–69 mg/dL, 3.0–3.8 mmol/L) and 0.0 [0.0, 0.0]% Level 2 TBR (<54 mg/dL, <3.0 mmol/L). All users met the clinical target of <4% time below 70 mg/dL (3.9 mmol/L) and <1% Level 2 TBR (<54 mg/dL, <3 mmol/L) over the aggregate 3‐month period [8]. A visual representation of TIR, TAR, and TBR for each age group is displayed in Figure 3.

**Figure 4:**
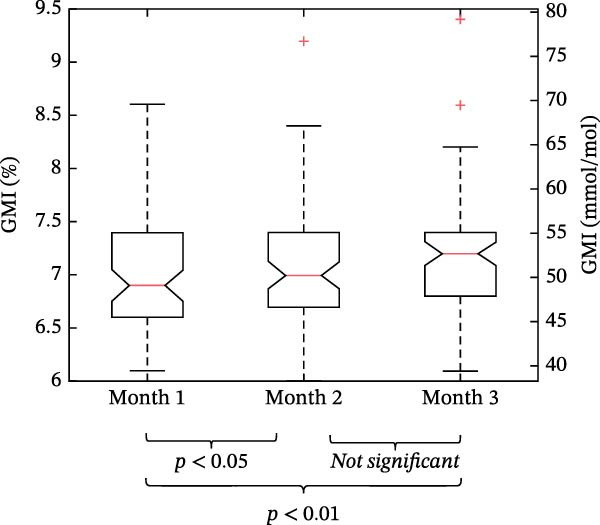
GMI in months 1–3 in the new‐onset T1D period among all Omnipod 5 users (*N* = 74). In the box plots, horizontal bars represent the median, the lower and upper boundaries of each box represent the 25^th^ and 75^th^ percentiles, and the plus signs represent outliers calculated by Q3 + 1.5  ^∗^ (Q3–Q1) for the upper fence and Q1−1.5  ^∗^ (Q3–Q1) for the lower fence.

Glycemic outcomes over each month of Omnipod 5 use in the new‐onset T1D period are displayed in Table 3. Median GMI was slightly lower in Month 1 (6.9 [6.6, 7.4]%) compared to Month 2 (7.0 [6.7, 7.4]%) and Month 3 (7.2 [6.8, 7.4]%) as shown in Figure 4. TIR was similar across each of the first 3 months of AID system use (*p* = 0.26). TBR remained low throughout the 3‐month study period, including Level 1 TBR: 1.0 [0.0, 1.0] % in Month 1, 0.0 [0.0, 1.0]% in Month 2, and 1.0 [0.0, 1.0] % in Month 3 and Level 2 TBR: 0.0 [0.0, 0.0]% in Month 1, 0.0 [0.0, 0.0]% in Month 2, and 0.0 [0.0, 0.0]% in Month 3.

**Table 3:** Real‐world glycemic outcomes by month in the initial 3 months of Omnipod 5 AID System use in the new‐onset T1D period (*N* = 74).

Glycemic outcomes	Month 1	Month 2	Month 3	*p*‐Value
GMI (%) GMI (mmol/mol)	7.0 ± 0.5 6.9 [6.6, 7.4] 53.9 ± 5.9 51.9 [48.6, 57.4]	7.1 ± 0.6 7.0 [6.7, 7.4] 53.7 ± 6.5 53.0 [49.7, 57.4]	7.2 ± 0.6 7.2 [6.8, 7.4] 54.6 ± 6.2 55.2 [50.8, 57.4]	*P* = 0.24

Level 2 time below range (%) <54 mg/dL [<3.0 mmol/L]	0.001 ± 0.12 0.0 [0.0, 0.0]	0.05 ± 0.23 0.0 [0.0, 0.0]	0.05 ± 0.23 0.0 [0.0, 0.0]	*p* = 0.35
Level 1 time below range (%) 54–69 mg/dL [3.0–3.8 mmol/L]	0.6 ± 0.6 1.0 [0.0, 1.0]	0.6 ± 0.8 0.0 [0.0, 1.0]	0.6 ± 0.7 1.0 [0.0, 1.0]	*p* = 0.86
Time in range (%) 70–180 mg/dL [3.9–10.0 mmol/L]	74.3 ± 14.6 75.0 [65.0, 85.0]	71.6 ± 15.1 72.0 [61.0, 82.0]	70.4 ± 14.2 69.0 [62.0, 81.0]	*p* = 0.26
Level 1 time above range (%) 181–250 mg/L [10.1–13.9 mmol/L]	17.9 ± 8.1 10.0 [13.0, 23.0]	19.4 ± 8.2 20.5 [14.0, 26.0]	19.9 ± 7.0 22.0 [16.0, 25.0]	*p* = 0.18
Level 2 time above range (%) >250 mg dL [>13.9 mmol/L]	7.2 ± 7.6 5.0 [1.0, 11.0]	8.4 ± 8.7 6.0 [2.0, 13.0]	9.0 ± 8.9 7.5 [3.0, 12.0]	*p* = 0.38

Coefficient of variation (%)	31.5 ± 5.7, 31.2 [28.3, 35.7]	31.8 ± 5.6, 31.9 [27.6, 35.5]	32.7 ± 5.6, 33.0 [29.4, 36.5]	*p* = 0.37

*Note:* Kruskal–Wallis rank sum test was used to analyze difference in glycemic metrics in months 1–3.

Glycemic outcomes were examined in more detail for users with an average glucose target of 110 mg/dL (*N* = 41) compared to those with a higher target than 110 mg/dL (*N* = 33). Among those using the lowest target of 110 mg/dL, GMI was lower (6.9 [6.7, 7.2]% vs. 7.3 [7.0, 7.6]%, *p* = 0.02), and TIR was higher (76.0 [67.8, 85.5]% vs. 67.0 [56.8, 75.5]%, *p* = 0.01). Additionally, 65.9% of those with an average target of 110 mg/dL achieved a TIR goal of >70% compared to 42.4% in the higher target group. There was a low percentage of hypoglycemia in users with the lowest target of 110 mg/dL, including 0.0 [0.0, 1.0]% Level 1 TBR and 0.0 [0.0, 0.0]% Level 2 TBR.

## 4. Discussion

This study provides a unique, retrospective analysis of youth with new‐onset T1D who initiated a tubeless AID system shortly after diagnosis. In the new‐onset T1D period, insulin needs fluctuate often. This can be attributed to the manifestation of the partial clinical remission phase, also known as the honeymoon phase, which is a transitory period marked by endogenous insulin production from surviving β‐cells following the introduction of insulin therapy [9, 10]. The Omnipod 5 AID System has proven effective at adjusting insulin delivery across diverse populations with established diabetes [11], but ours is the first study evaluating Omnipod 5 in the new‐onset T1D period. In this real‐world cohort, there were no episodes of DKA or severe hypoglycemia with AID system use in the observed new‐onset period. Total daily insulin usage over the initial 3 months of AID system use remained relatively stable for all age groups; however, body weight and C‐peptide levels were not available at each time point, so it was not possible to discern the extent of endogenous insulin production in the setting of partial clinical remission. Glycemic outcomes across the initial 3‐month period of AID system use were favorable, with a median GMI of 7.1 [6.7, 7.4]% (54.1 [49.7, 57.4] mmol/mol) and median TIR of 72.1 [64.0, 82.0]%. These results align closely with 2025 ADA Standards of Care for pediatric T1D management, which recommend an HbA1c of ≤7.0% (≤53 mmol/mol) and TIR >70% [12], indicating that early AID system use is associated with achieving glycemic targets.

The prevalence of hypoglycemia was low for this cohort of youth with new‐onset T1D using an AID system. For the aggregate 3‐month period of AID system use, the median Level 1 TBR was 1.0% and Level 2 TBR was 0%, both of which reside below the clinical target of <4% and <1%, respectively [12]. Notably, 96% of users had an overall time below the range of <70 mg/dL (<3.9 mmol/L) of <1%. These very low rates of hypoglycemia were present in those using a target setting of 110 mg/dL (6.1 mmol/L), which indicates the safety of using the lowest target setting with Omnipod 5. These outcomes are consistent with previous real‐world analyses of the Omnipod 5 AID System, indicating a pattern of low hypoglycemia risk in people with longer diabetes durations [11, 13]. Despite the potential hypoglycemia risk in the setting of partial clinical remission in the new‐onset period, initiation of Omnipod 5 was correlated with favorable glycemic patterns in this study.

Glycemic outcomes varied across age groups, with a lower GMI in the older pediatric age groups over the initial 3 months of Omnipod 5 AID System use in the new‐onset T1D period. 47.3% of the entire cohort achieved the 2025 ADA pediatric target GMI of ≤7.0% (≤53 mmol/mol) [12]. 76.2% of the oldest group (12 to <18‐year‐olds) met this target, while only 18.8% of the youngest group (0 to <6‐year‐olds) and 43.2% of 6 to <12‐year‐old achieved the target. Of note, the youngest group used higher target settings on their AID system and rarely used the lowest available target of 110 mg/dL (6.1 mmol/L), which was likely a key contributor to their higher GMI. The oldest group used the lowest available target setting of 110 mg/dL (6.1 mmol/L) most often, and their median GMI of 6.8% (50.8 mmol/mol) met the ADA goal, demonstrating optimal glycemic control in this age group.

One key outcome of this study was the variability in AID system target settings across age groups. The study included broad representation of the pediatric age range, and the youngest age group did include one participant under 2‐years‐old, which reflects the reality of off‐label use of Omnipod 5 in the real‐world clinical setting. The customizable glucose targets are an essential component of the Omnipod 5 AID System and can be leveraged in the new‐onset T1D period to accommodate fluctuating insulin needs. Providers and/or users can choose targets from 110 mg/dL to 150 mg/dL in 10 mg/dL increments (6.1 mmol/L to 8.3 mmol/L in 0.56 mmol/L increments) and set these targets in up to eight unique time segments throughout the day. The differences in target settings likely reflect the preference of diabetes providers, since initial pump settings and subsequent therapy adjustments are recommended by the provider, although this is often in alignment with family preferences. The youngest group (<6‐year‐olds) predominantly used higher glucose targets (130–150 mg/dL, 7.2–8.3 mmol/L), reflecting provider preference to minimize hypoglycemia risk in this population [3]. Parental involvement may also account for use of higher AID system target settings in young children, as parents of children with T1D commonly have fear of hypoglycemia [14–16]. During the 3 months post‐AID system initiation, the youngest group’s target values decreased from a median of 137 mg/dL (7.6 mmol/L) to 130.6 mg/dL (7.3 mmol/L). This gradual step‐down lines up with the spike‐and‐plateau pattern of parental fear of hypoglycemia reported in the recent‐onset T1D period [15]. The youngest group’s targets were consistently higher than the older age group’s, which both maintained median targets of 110 mg/dL (6.1 mmol/L) across all 3 months. This trend of lower target settings in older age groups might reflect the developmental shift in diabetes‐management responsibility from parents to youth. Adolescents and teenagers, who report lower fear of hypoglycemia than parents [16] and exercise more autonomy over pump settings [17], appear less inclined to choose higher targets. Overall, target flexibility with Omnipod 5 allows insulin delivery to be adjusted based on the unique needs and perceived risk profiles of different age groups, which may aid in optimizing glycemic outcomes while minimizing hypoglycemia risk.

In prior studies, use of the lowest target setting of 110 mg/dL with the Omnipod 5 AID System has been associated with lower HbA1c [3, 11]. In our analysis of youth with new‐onset T1D, over 60% of the cohort with an average glucose target of 110 mg/dL (6.1 mmol/L) achieved GMI ≤7% (≤53 mmol/mol) compared to 36.6% in those with a higher glucose target, indicating that use of the lowest target setting may be beneficial in achieving optimal glycemic control. Additionally, 66% of those with the lowest average target of 110 mg/dL achieved a TIR goal of >70% compared to 42% in the higher target group. Notably, the cohort using the lowest target setting demonstrated minimal hypoglycemia risk with a median of 1% Level 1 TBR and 0% Level 2 TBR.

Intensive insulin therapy is a well‐documented factor in reducing long‐term T1D complications [18]. The new‐onset period is particularly significant, and early implementation of intensive insulin therapy leads to a reduction in the micro‐ and macrovascular complications [1]. The glycemic benefit of Omnipod 5 during the new‐onset period suggests that early initiation of AID systems may play a key role in mitigating long‐term complication risk in people with T1D. Although it was not statistically significant, there was a trend toward decreasing TIR and increasing TAR over time during the first 3 months of AID system use in the new‐onset period. It is unclear if this was due to waning partial remission, and future studies with longer duration are needed to determine if the initial impact of AID system use has longer‐term benefits in decreasing micro‐ and macrovascular complications.

Although our institution routinely presents all commercially available AID systems to families during the new‐onset education process, this study focused specifically on the Omnipod 5 AID System. This approach was intentional for two reasons. First, during the study period, Omnipod 5 was the only AID system available through the pharmacy channel in the U.S., which enabled rapid procurement, streamlined training, and facilitated early initiation shortly after diagnosis. Second, Omnipod 5 was the only AID platform with automatic, cloud‐based data transmission to Glooko, the device data management system used for clinical decision‐making and research at our institution. These characteristics ensured consistent, complete, and reliable data capture across all participants included in this analysis. Broader, prospective evaluation of early AID system use across multiple device platforms is warranted, and future studies should examine whether clinical outcomes vary by AID technology during the new‐onset period.

This study has several important limitations. First, its retrospective design and lack of a control group limit the ability to infer causality between early AID system use and observed glycemic outcomes. Second, the relatively short 3‐month follow‐up period may not capture the full trajectory of insulin needs, glycemic patterns, or safety outcomes as the partial clinical remission phase evolves. Third, selection bias is possible, as families who elect to initiate an AID system soon after diagnosis may differ in comfort with diabetes technology or engagement with diabetes care compared with the broader newly diagnosed population. Fourth, glycemic metric analysis relied on CGM‐derived metrics from Glooko, which did not provide ADA time in a tight range (70 mg/dL–140 mg/dL, 3.9–7.8 mmol/L), and laboratory HbA1c values were not consistently available at the relevant time points, requiring use of GMI as an estimate. Finally, body weight, C‐peptide levels, and other clinical markers of endogenous insulin production were not consistently available, preventing assessment of the degree to which partial clinical remission contributed to insulin requirements or glycemic outcomes. Despite these limitations, this study provides real‐world evidence on the safety and glycemic impact of AID system use in the new‐onset T1D period.

## 5. Conclusion

In this real‐world observational study of youth with new‐onset T1D using the Omnipod 5 AID System, there were no episodes of DKA or severe hypoglycemia, indicating the safety of AID system use directly after T1D diagnosis. Future studies should assess whether early adoption of AID systems impacts long‐term glycemic control and diabetes‐related quality of life.

## Funding

This study was funded by an investigator‐initiated award from Insulet.

## Disclosure

Preliminary findings from this study were presented in a poster at the International Society of Pediatric and Adolescent Diabetes (ISPAD) 50^th^ Annual Conference in October 2024. The authors had full autonomy in the study design, execution and analysis, manuscript conception, planning, writing, and decision to publish. The funder had no role in the design, data collection, data analysis, and reporting of this study.

## Ethics Statement

This study protocol was reviewed and approved by the Baylor College of Medicine Institutional Review Board for Human Subject Research (BCM IRB), which granted a waiver of consent, Approval Number [H‐54814]. The BCM IRB is organized, operates, and is registered with the United States Office for Human Research Protections according to the regulations codified in the United States Code of Federal Regulations at 45 CFR 46 and 21 CFR 56. The BCM IRB operates under the BCM Federal Wide Assurance Number 00000286.

## Conflicts of Interest

Daniel J. DeSalvo reports research support from Insulet to his institution during the conduct of the study, and he has received consultant fees from Dexcom, Insulet, MannKind, and Sanofi outside the submitted work. Marzia Cescon serves on the advisory board for Diatech Diabetes, Inc. The other authors declare no conflicts of interest.

## Data Availability

The data that support the findings of this study are available upon request from the corresponding author. The data are not publicly available due to privacy or ethical restrictions.
